# An Improved Method to Compute the Mutual Capacitance between Interdigital Transducers in Radio Frequency Surface Acoustic Wave Filters

**DOI:** 10.3390/mi15050661

**Published:** 2024-05-18

**Authors:** Yali Zou, Xinyu Yang, Ping Luo, Yuhao Liu

**Affiliations:** 1 School of Microelectronics, Shanghai University, Shanghai 200444, China; yuhaoliu@shu.edu.cn; 2 Changzhou ChemSemi Co., Ltd., Changzhou 213164, China

**Keywords:** RF filter, surface acoustic wave, interdigital transducer, DMS, mutual capacitance

## Abstract

This paper proposes an improved method to calculate the mutual capacitance between interdigital transducer (IDT) electrodes to enhance the accuracy of the traditional coupling-of-modes (COM) model, which is commonly used to simulate surface acoustic wave (SAW) filters and duplexers. In this method, the boundary element method (BEM) is adopted to obtain the capacitance per unit length in a layered medium, while the partial capacitance (PC) method is used to derive the effective relative permittivity of the multi-layered IDT. Numerical results from commercially available software are provided for comparison with the results calculated using the proposed method. The consistent results verify the validity and accuracy of this method, which also demonstrates significantly faster calculation speed compared to commercially available software. Precise electrical response prediction of a dual-mode SAW (DMS) filter can be achieved by applying this method to the COM model, and this ultra-fast calculation method can also be included in filter design optimization.

## 1. Introduction

SAW filters, known for their compact size and superior out-of-band rejection capabilities, are extensively utilized in the radio frequency front-ends (RFFE) of mobile communication systems. Although SAW devices exhibit exceptional performance in mobile communications, there is an increasing need to enhance the resonance quality factor further [1]. The typical configurations of SAW filter circuits encompass ladder-type circuits, DMS (dual-mode SAW) type circuits, and hybrid ladder and DMS type circuits. DMS filter circuits are distinguished by their low insertion loss, compact chip size, and excellent out-of-band suppression. When compared to ladder-type filter circuits, which occupy a larger area but offer higher input power tolerance, DMS type filter circuits are typically preferred for applications in the receive path of a duplexer or a single receive band filter. DMS filter circuits often exhibit clean dual-mode resonances along with multiple out-of-band resonances, which can manifest as unwanted spurious modes within the rejection band. To ensure successful duplexer design, these unwanted spurious modes must be accurately captured by the simulation model and subsequently optimized out during the duplexer simulation iterations. Consequently, the precision of the DMS circuit model and the speed of calculation are of paramount importance for the design of the filter.

Accurate and efficient modeling of surface acoustic wave (SAW) resonators is indispensable for the design and optimization of high-performance SAW filters [2,3,4]. A plethora of models have been proposed to facilitate the analysis of SAW devices, often by simplifying the intricate physical aspects of these devices. These models encompass the impulse model [5], equivalent circuit models [6], the coupling-of-modes (COM) model [7], the P-matrix model [8], and the scattering matrix approach [9]. In addition to these models, finite element methods (FEMs) [10,11] and boundary element methods (BEMs) [12,13,14] are also extensively employed for the analysis of SAW devices.

The finite element method (FEM) offers unique advantages in simulations, including its capability to model spurious modes [15], nonlinear effects [16], and SAW devices with complex geometries [17]. Conversely, BEM provides an exact solution for the system and offers several efficiency advantages through direct full-wave analysis based on the fundamental wave equation and boundary conditions. In hybrid FEM/BEM simulations [18,19], FEM is typically employed to simulate arbitrarily shaped finger electrodes, while BEM is utilized to simulate the semi-infinite piezoelectric substrate. This combined approach leverages the strengths of both methods, ensuring a comprehensive and accurate analysis of SAW device behavior.

The coupling-of-modes (COM) model is the most widely utilized approach for computing the electrical response of SAW filters, as it strikes an optimal balance between calculation speed and accuracy. However, the standard COM model primarily focuses on discrete interdigital transducer (IDT) sections and does not fully account for the mutual capacitive coupling between IDTs. This limitation can result in an inaccurate representation of the out-of-band electrical response.

**Figure 1:**
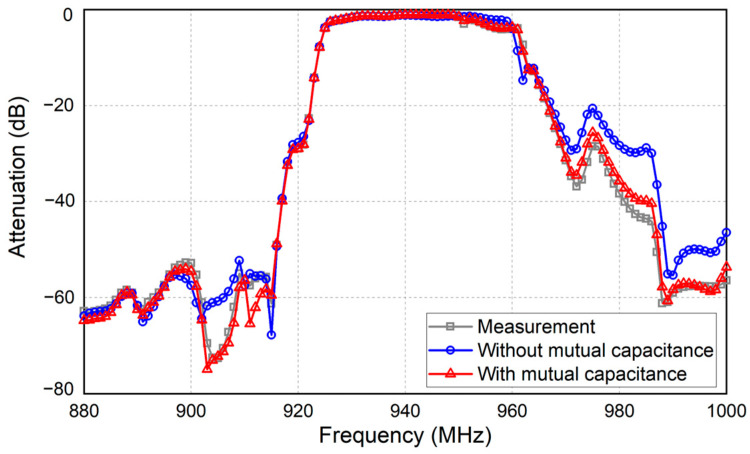
Electrical responses of the DMS filters with and without considering mutual capacitance compared with the measurement result.

As illustrated in Figure 1, the mutual capacitance has a significant impact on the out-of-band attenuation performance, particularly in the lower side rejection of a dual-mode SAW (DMS) filter. To enhance the precision of the COM model in such scenarios, it is crucial to refine the model to include these previously overlooked mutual capacitance effects.

To enhance the accuracy of the model, one viable solution is to incorporate the mutual capacitance between IDT electrodes across different acoustic tracks within the COM model. Typically, lumped capacitors are added between IDTs to simulate the effects of mutual capacitance. However, the values of these lumped capacitors often need to be determined through experimental data, which can be labor-intensive and may not provide a complete theoretical understanding.

An alternative approach involves utilizing the finite element method (FEM), which is generally acknowledged as the most authentic simulation technique, to calculate mutual capacitance. While this method can yield accurate results, it is computationally intensive and may not be practical for implementation in filter optimization processes due to the significant time required for calculations.

Therefore, this study presents a novel approach that employs the boundary element method (BEM) and the partial capacitance (PC) method to enable rapid and accurate prediction of mutual capacitance. For validation, the mutual capacitance between a periodic IDT with ten finger electrodes, fabricated on a 128°YX-cut LiNbO_3_ substrate, is calculated using both the proposed method and FEM. The results from the two techniques are then compared and discussed. Furthermore, by integrating the COM model, the researchers have designed a high-performance, temperature-compensated SAW (TC-SAW) duplexer. This duplexer incorporates a DMS filter in the receive path, achieving precise out-of-band rejection and isolation.

## 2. Materials and Methods

**Figure 2:**
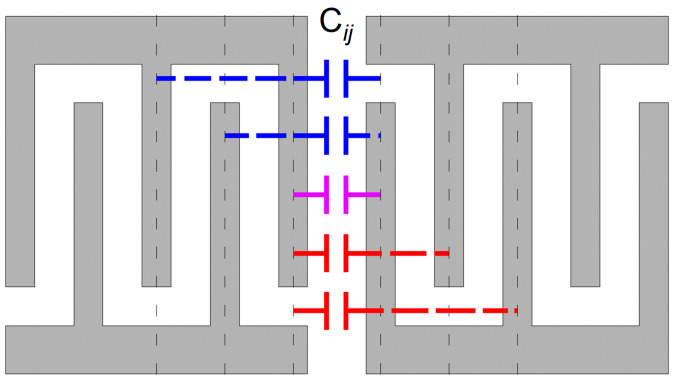
Diagram of the IDT showing capacitance between fingers. Purple line is the capacitor between the adjacent fingers. Red dash lines are the capacitors between the last finger of the left IDT and the further fingers of the right IDT. Blue dash lines are the capacitors between the first finger of the right IDT and the further fingers of the left IDT.

Building on the introduction, the researchers conduct a comprehensive analysis and calculation of the mutual capacitance between IDTs in the structure of DMS-type filters. In the conventional COM model, only the capacitance between adjacent electrodes of two neighboring IDT tracks is considered, as depicted by the solid line capacitors in Figure 2. For more complex duplexer designs that require higher precision, it is necessary to consider the capacitance between electrodes that are further apart, as indicated by the dashed line capacitors in Figure 2. Incorporating these additional capacitance elements is essential for improving the model’s accuracy in predicting duplexer isolation, which is typically expected to be in the range of −60 to −70 dB.

**Figure 3:**
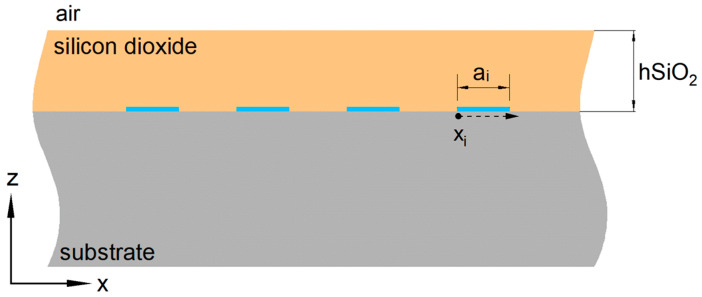
Cross-section of the periodic IDT with two dielectric layers.

As a case study, we focus on the basic DMS structure of a TC-SAW device, using LiNbO_3_ as the substrate. The cross-section of a periodic IDT with two dielectric layers above the electrode layer is shown in Figure 3. Relative permittivity of each dielectric layer is denoted as $\varepsilon_{1}$ and $\varepsilon_{2}$, respectively. The first dielectric adjacent to the electrodes has a finite thickness. The thickness of the second dielectric referring to air in our case is infinite. The electrodes of IDT have an aperture of W and a width of $a_{i}$ for the *i*-th electrode. And $x_{i}$ indicates the relative *x* position from the left side of *i*-th electrode.

The following proposed method in this study is based on three fundamental assumptions: (1) that the electrode thickness is significantly smaller than both the aperture and the electrode width, allowing the electrodes to be treated as sheet conductors in the calculation; (2) that the substrate thickness is generally much larger than the overlying layers, and is therefore considered infinite for the purposes of this analysis; and (3) that the dielectric layer length in the x-direction is assumed to be infinite, a common approximation in the analysis of periodic structures.

### 2.1. Capacitance Matrix of Finger Electrodes

The boundary element method (BEM) has gained widespread adoption for modeling SAW devices. By employing orthogonal function expansions on electrodes, BEM achieves accurate calculations at the electrode edges. Building upon this foundation, this paper introduces an improved method based on BEM for computing the mutual capacitance between electrodes of neighboring IDTs with a high degree of accuracy and in significantly less time than traditional FEM analyses. To address the singularity at the edges of the electrodes, the proposed method utilizes BEM with weighted Chebyshev polynomials of the first kind as the expansion functions [20]. The upper limit of the order of Chebyshev polynomials, described as K, is an integer greater than zero. With increasing K value, the calculating results associated with electrical charges are becoming more accurate.

Considering the singularity on both edges of electrodes, the first kind Chebyshev polynomials weighted by a reciprocal of the square root are used as basic functions. Surface charge distribution on the *i*-th electrode can then be written as the following series expansion functions:

(1) $$\sigma_{i}(x)=\sum_{j=0}^{K}A_{ij}\frac{T_{j}(u)}{\sqrt{1-u^{2}}},\;x_{i}-\frac{a_{i}}{2}<x<x_{i}+\frac{a_{i}}{2}$$

where $u=2(x-x_{i})/a_{i}$, $T_{j}$ is the *j*-th order Chebyshev polynomials of the first kind, $x_{i}$ is the center of the *i*-th electrode, and $A_{ij}$ are undetermined coefficients. The charge distribution on each electrode will only be determined if coefficients $A_{ij}$ are derived.

The electrical potential of infinite periodic IDT is described as an integration of charge distribution:

(2) $$\phi (x)=\int_{-\infty }^{+\infty }G(x-x’)\sigma (x’)dx’$$

where G(x) is the Green’s function, which has been given in [20]. Substituting Equation (1) into Equation (2), the electrical potential distribution in the x-axis is then given as follows:

(3) $$\phi (x)=\sum_{i=1}^{M}\sum_{j=0}^{K}A_{ij}\frac{a_{i}}{2}\int_{-1}^{1}G(x+x_{i}-\frac{a_{i}}{2}u)\frac{T_{j}(u)}{\sqrt{1-u^{2}}}du$$

where M is the total number of IDT electrodes. The charge distributions are solved from Equations (1) and (3) by giving ϕ(x) for M·(K+1) discrete sampling points on electrodes. For simplification, we specify K as an even number. If $K=0,\;2,\;4,\;\cdots ,\;k_{n}$, suggested x coordinates of the sampling points within each electrode are list in Table 1. $\sigma_{i}(x)$ becomes more accurate with larger K order of Chebyshev polynomials. The total charge of *i*-th electrode is computed by integration over width according to the following expression:

(4) $$Q_{i}=\int_{x_{i}-\frac{a_{i}}{2}}^{x_{i}+\frac{a_{i}}{2}}\sigma_{i}(x)d(x)$$

When a specific voltage is applied to only one electrode and all other electrodes are grounded, the capacitance related to this electrode can be determined by measuring the charges. Taking $V_{i}$ as the voltage applied to each electrode, capacitance per unit length in vacuum, $C_{ij}$, is obtained by the following simultaneous linear equations, in matrix form:

(5) $$\left[\begin{array}{c} Q_{1}\\ Q_{2}\\ \vdots \\ Q_{M} \end{array}\right]=\left[\begin{array}{cccc} C_{11} & C_{12} & \ldots & C_{1M}\\ C_{21} & C_{22} & \ldots & C_{2M}\\ \vdots & \vdots & \ddots & \vdots \\ C_{M1} & C_{M2} & \ldots & C_{MM} \end{array}\right]\left[\begin{array}{c} V_{1}\\ V_{2}\\ \vdots \\ V_{M} \end{array}\right]$$

Considering the multi-layered substrate and aperture of IDT, final capacitance matrix $C_{f}$ is obtained:

(6) $$C_{f}=\left[\begin{array}{cccc} C_{11} & C_{12} & \ldots & C_{1M}\\ C_{21} & C_{22} & \ldots & C_{2M}\\ \vdots & \vdots & \ddots & \vdots \\ C_{M1} & C_{M2} & \ldots & C_{MM} \end{array}\right]\cdot W\cdot \varepsilon_{eff}$$

where $\varepsilon_{eff}$ is the relative effective permittivity for multi-layered dielectrics above and below the electrodes.

### 2.2. Relative Effective Permittivity

The partial capacitance (PC) method, initially proposed by Kochanov [21], offers an alternative. It was originally applied to coplanar waveguides on substrates with finite thickness. When combined with the conformal mapping technique, the PC method has been utilized to model interdigital capacitors with multi-layered structures [22,23]. Although conformal mapping can deliver precise results, it is often constrained by the complexity of the model’s geometry, which can make it challenging to apply in practical design scenarios. The partial capacitance (PC) method is applied to the calculation of mutual interdigital capacitors on multi-layered substrates [24].

The relative permittivity above electrodes is computed from lower to upper dielectric iterative by applying Green’s function [25]. In our case, $\mathrm{Si}O_{2}$ has a height of $h_{\mathrm{Si}O_{2}}$:

(7) $$\varepsilon_{above}=\varepsilon_{1}\cdot \frac{\varepsilon_{1}\tanh\left(\frac{2\pi }{\lambda }h_{\mathrm{Si}O_{2}}\right)+\varepsilon_{2}}{\varepsilon_{1}+\varepsilon_{2}\tanh\left(\frac{2\pi }{\lambda }h_{\mathrm{Si}O_{2}}\right)}$$

For a hexagonal piezoelectric material below electrodes, the relative permittivity is given by the following [26]:

(8) $$\varepsilon_{below}=\varepsilon_{0}+\sqrt{\varepsilon_{11}^{T}\varepsilon_{33}^{T}-\varepsilon_{13}^{T2}}$$

where $\varepsilon_{11}$ and $\varepsilon_{33}$ are the principal dielectric constant, and the superscript T indicates that the permittivities are measured under the zero-stress condition. Under the low-frequency approximation, the relative effective permittivity is simply given as parallel connection by the following:

(9) $$\varepsilon_{eff}=\varepsilon_{above}+\varepsilon_{below}$$

### 2.3. Mutual Capacitance of DMS Filters

In this case, the measured capacitance matrix is defined as the Maxwell capacitance, which is, however, not convenient to implement in a typical circuit simulator. A mutual capacitance matrix form between discrete electrodes is used instead:

(10) $$\left[\begin{array}{cccc} C_{m,11} & C_{m,12} & \ldots & C_{m,1M}\\ C_{m,21} & C_{m,22} & \ldots & C_{m,2M}\\ \vdots & \vdots & \ddots & \vdots \\ C_{m,M1} & C_{m,M2} & \ldots & C_{m,MM} \end{array}\right]=\left[\begin{array}{cccc} \sum_{i=1}^{M}C_{1i} & -C_{12} & \ldots & -C_{1M}\\ -C_{21} & \sum_{i=1}^{M}C_{2i} & \ldots & -C_{2M}\\ \vdots & \vdots & \ddots & \vdots \\ -C_{M1} & -C_{M2} & \ldots & \sum_{i=1}^{M}C_{Mi} \end{array}\right]$$

where $C_{m,ij}$ in the matrix stands for mutual capacitance elements.

**Figure 4:**
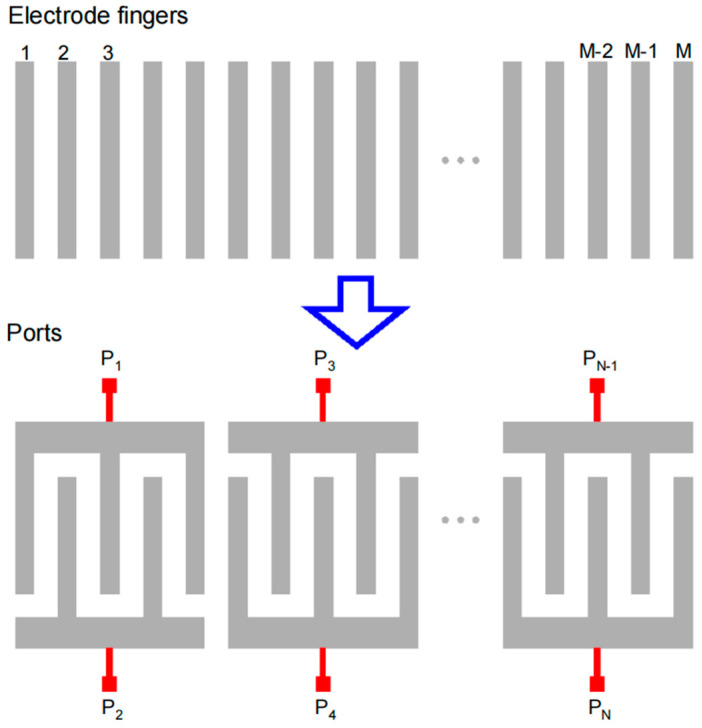
Diagram of the reduction in the dimension of capacitance matrix. Number of *M* electrode fingers, arranged in gratings, are connected by *N* ports in the DMS filter simulation.

As shown in Figure 4, when a practical DMS filter is simulated, periodic finger electrodes are classified into an even number of groups according to IDT ports. Finger electrodes with the same port are paralleled connected, resulting in the same electrical potential, which indicates zero capacitance within the same port. As a result, the dimension of the capacitance matrix obtained from BEM is reduced from M×M to N×N for N-port DMS filter simulation. The diagonal elements in the matrix are self-capacitance, and capacitance between the facing ports needs to be set to zero due to the intrinsic value form COM model. The capacitance matrix of the IDT ports is convenient to integrate into a circuit simulator once it is computed.

By integrating this advanced approach into the conventional COM model, a mutual capacitance matrix is introduced, resulting in a modified model that can accurately predict the out-of-band electrical responses of dual-mode SAW (DMS) filters.

## 3. Results and Discussion

### 3.1. Simulation of One Pair of IDTs

Building on the analysis presented in the previous section, the capacitance between the electrodes of a multi-layered dielectric IDT was computed. The results were subsequently compared with those obtained from a commercial finite element method (FEM) solver. The periodic IDT electrode pitch was set at 2 μm with varying duty factors (DF) of 0.3, 0.5, and 0.7, respectively. A 1.2 μm thick layer of SiO_2_ was deposited on a 128°YX-cut lithium niobate (LiNbO_3_) piezoelectric substrate to serve as a temperature compensation layer, enveloping the electrodes. The number of finger electrodes is 10, denoted as M=10. To enhance the accuracy of the results, the order of the Chebyshev polynomials, K, was set to 2, indicating that three discrete sampling points along each electrode were required in the calculation. Simulation parameters are summarized in Table 2. This results in a 10×10 matrix being solved by the proposed method.

**Figure 5:**
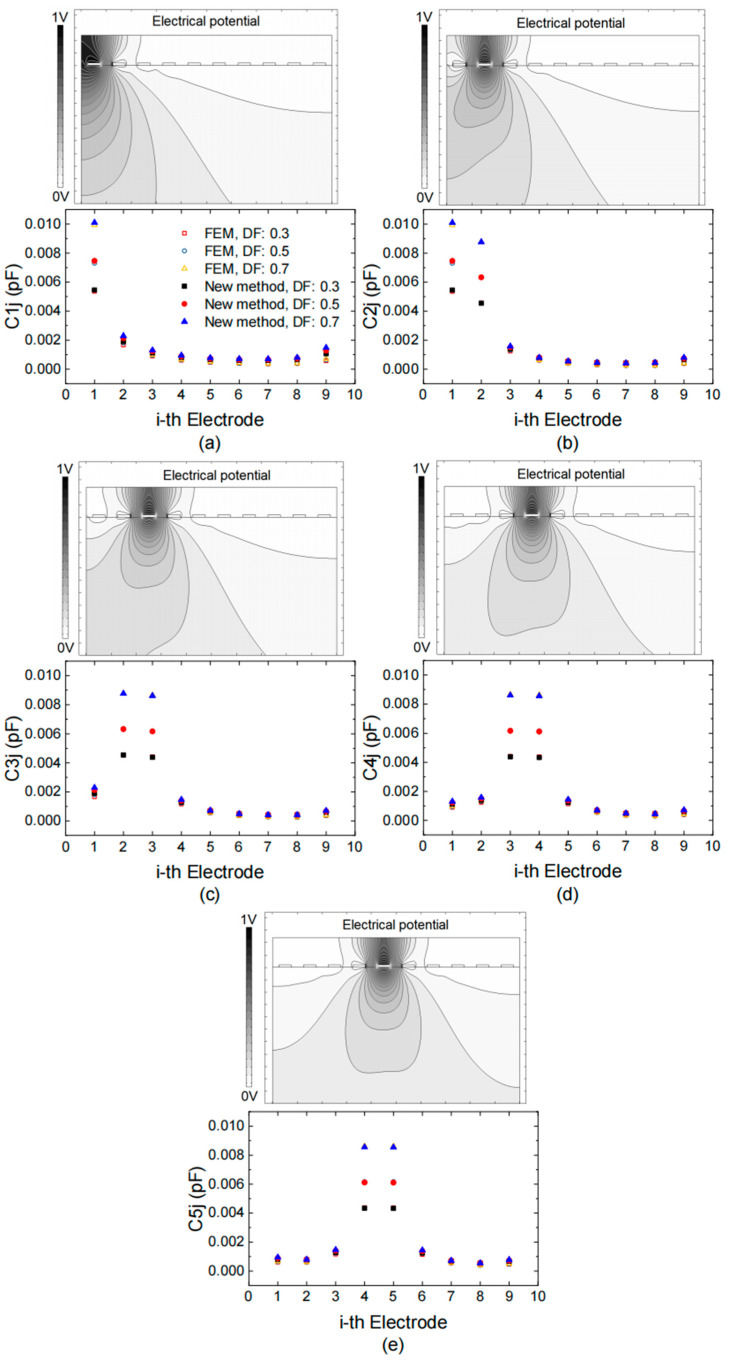
Electrical potential distribution within the stack (upper) and capacitance evaluated by new method and commercial FEM solver (lower) when 1V voltage is applied on (**a**) the first electrode, (**b**) the second electrode, (**c**) the third electrode, (**d**) the fourth electrode and (**e**) the fifth electrode, respectively.

**Figure 6:**
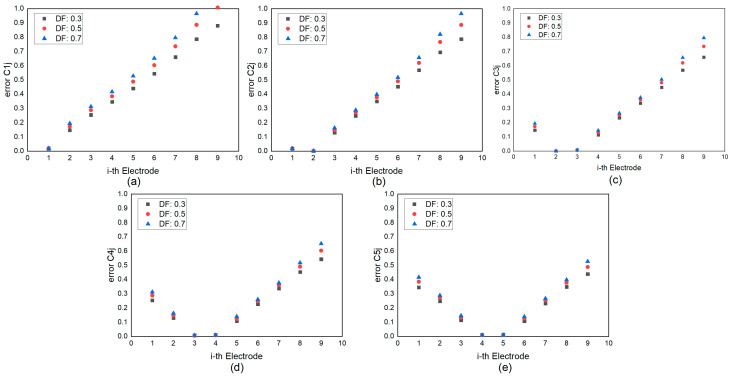
Dependence of the calculation error in capacitance on the *i*-th electrode when 1V voltage is applied on (**a**) the first electrode, (**b**) the second electrode, (**c**) the third electrode, (**d**) the fourth electrode and (**e**) the fifth electrode, respectively.

The mutual capacitance for 10 finger electrodes was calculated using both our modified method and a commercial finite element method (FEM) solver. Due to the symmetric conformation, only half of the capacitance is shown in Figure 5. Figure 5a–e illustrate the electrical potential distribution within the stack and provide a comparison of the capacitance values obtained from the two methods, respectively. The capacitance computed by the modified method closely aligns with the results from the commercial FEM solver, with slightly greater differences at the edges compared to the center due to fringing effects accounted for in the FEM simulation. The error associated with the current method was assessed [27,28]. For a given $\varepsilon_{eff}$ value, the error increases monotonically as the spacing between fingers increases. A value less than 0.5 appears to be reasonable when modeling TC-SAW IDTs, considering the capacitance magnitude is on the order of $10^{-4}$, which minimally impacts filter performance. Figure 6 illustrates the estimated error in capacitance related to the *i*-th electrode, providing guidance that mutual capacitance beyond five adjacent fingers can be disregarded in the simulation of TC-SAW DMS filters.

The FEM solver required 41 s to compute a capacitance matrix using a server equipped with 52 cores and 104 threads. In contrast, the proposed method achieved the same computation in just 0.62 s for the same structure, representing a remarkable 98.5% reduction in simulation time compared to FEM. Relative to the FEM, this substantial enhancement in simulation efficiency allows for a higher iteration frequency during the design process of filters and significantly reduces the time cost associated with filter optimization. Consequently, the proposed method offers the potential to achieve precise design and optimization of dual-mode SAW (DMS) filters, facilitating more efficient and effective filter development.

### 3.2. Design of a TC-SAW Duplexer

**Figure 7:**
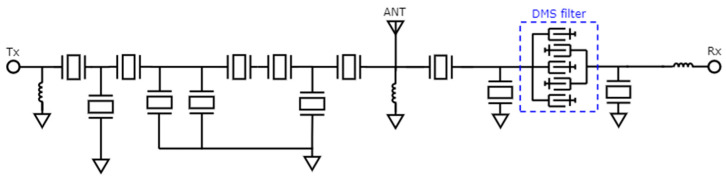
Designed topology of a 5G New Radio (NR) Band VIII TC-SAW duplexer. Tx filter is a pure ladder-type filter. Rx filter is composed of resonators and a DMS filter.

**Figure 8:**
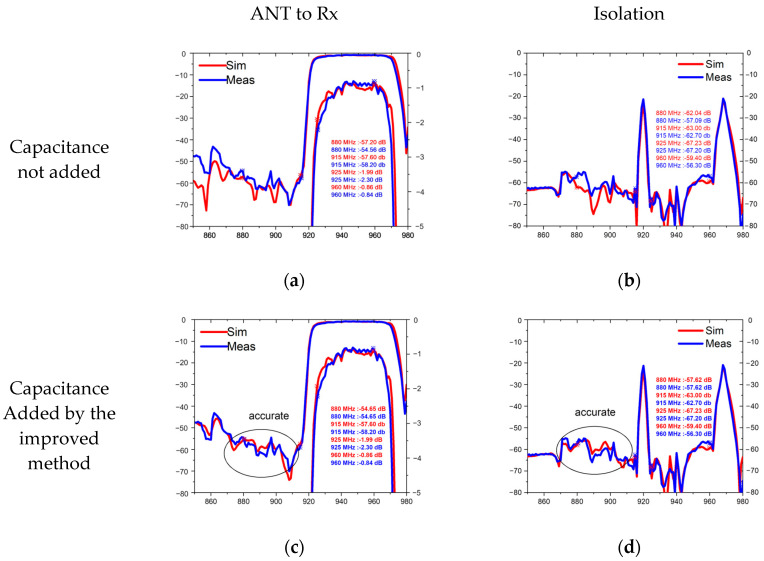
Comparison of simulated and measured with and without capacitance. (**a**) Antenna-to-Rx comparison without mutual capacitance; (**b**) isolation without mutual capacitance; (**c**) comparison of results from antenna to Rx with added mutual capacitance; (**d**) comparison of isolation after adding the mutual capacitor.

To confirm the efficacy of the proposed method, it was integrated into the conventional COM model and utilized to design and optimize a 5G New Radio (NR) Band VIII TC-SAW duplexer. Figure 7 shows the topology of the Band VIII duplexer, where the transmitting (Tx) filter is a pure ladder-type filter, while the receiving (Rx) filter is composed of resonators as well as a DMS. The inclusion of the DMS filter in the Rx filter is essential due to its superior out-of-band rejection and isolation, particularly on the lower side of frequency responses [29,30,31]. The NR Band VIII duplexer was fabricated using a high-Q TC-SAW process in Changzhou ChemSemi Corporation (Changzhou, China) [32]. Figure 8 presents a comparative analysis of the simulation and experimental results for the NR Band VIII duplexer, with the red line indicating the simulation outcomes and the blue line representing the measured data. Figure 8a,c depict the S21 transmission loss from the antenna to the Rx, while Figure 8b,d illustrate the duplexer’s isolation performance.

Comparing the simulated and measured results, both with and without accounting for mutual capacitance, reveals that the proposed method yields a superior fit between simulation and experimental outcomes, particularly in the prediction of critical isolation performance. This underscores the importance of accurate models. In the practical design of a duplexer, the IDTs of DMS filters in the Rx path are unlikely to achieve perfectly periodic conditions. Particularly in the regions near the adjacent IDT fingers, the pitch often undergoes substantial variations [33]. With this improved model, the effects of non-periodic IDT pitch can be more rapidly and accurately captured by adjusting the derived parameters $x_{i}$ and $a_{i}$.

Table 3 provides a comparative analysis of the present method against two other techniques, highlighting the distinct characteristics and performance of each method. According to the table, all three methods are capable of meeting the accuracy requirements for the SAW model. The finite element method (FEM) is notable for its high accuracy but is also characterized by its extremely slow computation speed. Conversely, the conformal mapping method, while offering a certain level of accuracy, is more complex in its implementation. The method proposed in this paper stands out for its ability to maintain sufficient accuracy without increasing the complexity of the model and for its significant improvement in computational speed. This efficiency places the proposed method ahead of the other techniques in terms of overall performance, offering a superior approach for the design and optimization of SAW filters and duplexers.

## 4. Conclusions

This work presents an effective method that combines the BEM and the PC methods to obtain the mutual capacitance matrix of IDT electrodes with multi-layered dielectrics. The mutual capacitance of ten periodic IDT electrodes was computed using the proposed method, and the results were found to be in good agreement with those calculated from a commercial FEM solver. The application of this method enables the precise prediction of the out-of-band rejection of DMS filters while significantly reducing the computation time required. This enhanced efficiency allows for the seamless integration of the proposed method into the filter optimization process. The ability to accurately model the mutual capacitance between IDT electrodes is crucial for the design of high-performance SAW and TC-SAW filters, particularly those incorporating DMS structures, targeting 5G New Radio applications.

## Figures and Tables

**Table 1:** Sampling points of solving the charge distribution with different *K*.

K	x-Axis Coordinate of Sampling Points
0	$x_{i}+\frac{a_{i}}{2}$
2	${x_{i}\;,\;x}_{i}+\frac{a_{i}}{2},\;{\;x}_{i}+a_{i}$
4	${x_{i}\;,\;x}_{i}+\frac{a_{i}}{4},\;{\;x}_{i}+\frac{a_{i}}{2}\;,\;{\;x}_{i}+\frac{{3a}_{i}}{4}\;,{\;x}_{i}+a_{i}$
⋯	⋯
$k_{n}$	${x_{i}\;,\;x}_{i}+\frac{a_{i}}{k_{n}},\;{\;\cdots ,\;x}_{i}+\frac{\left(k_{n}-1\right)a_{i}}{k_{n}}\;,\;{\;x}_{i}+a_{i}$

**Table 2:** Simulation parameters table.

Simulation Parameters	Value
Number of fingers (*M*)	10
Duty factors (DF)	0.3/0.5/0.7
Pitch	2 μm
Aperture (*W*)	16 × pitch
$\mathrm{Thickness}\;\mathrm{of}\;{\mathrm{SiO}}_{2}\;(h_{{\mathrm{SiO}}_{2}}$)	1.2 μm
Order of Chebyshev polynomials (*K*)	2

**Table 3:** Summary of the improved method and other methods.

Method	FEM	Conformal Mapping	Capacitance Added by the Improved Method in This Paper
Effect	Accuracy of SAW models can be achieved		
Pros and cons	1. High accuracy 2. Extremely slow speed	1. Moderate accuracy 2. Complex models	1. Precise and ultra-fast computation 2. Contribute to filter optimization

## Data Availability

The original contributions presented in the study are included in the article, further inquiries can be directed to the corresponding author.

## References

[1] Wu J., Zhang S., Zhang L., Zhou H., Zheng P., Yao H., Li Z., Huang K., Wu T., Ou X. (2022). Exploring Low-Loss Surface Acoustic Wave Devices on Heterogeneous Substrates. IEEE Trans. Ultrason. Ferroelectr. Freq. Control.

[2] Hagelauer A., Fattinger G., Ruppel C.C.W., Ueda M., Hashimoto K.-Y., Tag A. (2018). Microwave acoustic wave devices: Recent advances on architectures, modeling, materials, and packaging. IEEE Trans. Microw. Theory Tech..

[3] Nakamura H., Komatsu T., Nakanishi H., Tsurunari T., Fujiwara J. Reduction of transverse leakage for SAW resonators on LiTaO_3_ substrate. Proceedings of the IEEE International Ultrasonics Symposium 2012.

[4] Wu Z., Liu Y.-M., Shi B., Bao J.-F., Hashimoto K.-Y. COM-based modeling of saw scattering at reflector outer edges in IHP SAW resonator. Proceedings of the IEEE International Ultrasonics Symposium 2022.

[5] Hartmann C.S., Bell D.T., Rosenfeld R.C. (1973). Impulse model design of acoustic surface-wave filters. IEEE Trans. Microw. Theory Tech..

[6] Hoang T., Beghi M.G. (2011). SAW parameters analysis and equivalent circuit of SAW device. Acoust. Waves-Microdevices Helioseismology.

[7] Plessky V., Koskela J. (2000). Coupling-of-modes analysis of SAW devices. Int. J. High Speed Electron. Syst..

[8] Kanouni F., Amara S., Assali A., Arab F., Qin Z. (2020). A P-matrix-based model for the frequency analysis of IDT/AlScN/Sapphire SAW-delay line. Sens. Actuators A Phys..

[9] Soluch W. (2000). Scattering matrix approach to one-port SAW resonators. IEEE Trans. Ultrason. Ferroelectr. Freq. Control.

[10] Hofer M., Finger N., Kovacs G., Schoberl J., Zaglmayr S., Langer U., Lerch R. (2006). Finite-element simulation of wave propagation in periodic piezoelectric SAW structures. IEEE Trans. Ultrason. Ferroelectr. Freq. Control.

[11] Chamaly S., Fong H.Y., Perois X., Mayer M. Very low amplitude ripple SAW filter for infrastructure systems using 41° YX lithium niobate: Full FEM/BEM design approach. Proceedings of the IEEE International Ultrasonics Symposium 2009.

[12] Qiao D., Liu W., Smith P.M. (1999). General Green’s functions for SAW device analysis. IEEE Trans. Ultrason. Ferroelectr. Freq. Control.

[13] Fu Q., Luo W., Wang Y., Wang J., Zhou D. Simulation of wireless passive SAW sensors based on FEM/BEM model. Proceedings of the IEEE Ultrasonics Symposium 2008.

[14] Benes E., Groschl M., Seifert F., Pohl A. (1998). Comparison between BAW and SAW sensor principles. IEEE Trans. Ultrason. Ferroelectr. Freq. Control.

[15] Xu H., Fu S., Shen J., Lu Z., Su R., Wang R., Song C., Zeng F., Wang W., Pan F. (2022). Large-range spurious mode elimination for wideband SAW filters on LiNbO_3_/SiO_2_/Si Platform by LiNbO_3_ cut angle modulation. IEEE Trans. Ultrason. Ferroelectr. Freq. Control.

[16] Li B., Zhang Q., Zhao X., Zhi S., Qiu L., Fu S., Wang W. (2022). A general FEM model for analysis of third-order nonlinearity in rf surface acoustic wave devices based on perturbation theory. Micromachines.

[17] Li X., Bao J., Huang Y., Zhang B., Omori T., Hashimoto K.-Y. (2019). Use of hierarchical cascading technique for FEM analysis of transverse-mode behaviors in surface acoustic-wave devices. IEEE Trans. Ultrason. Ferroelectr. Freq. Control.

[18] Solal M., Chen L., Gratier J. (2013). Measurement and FEM/BEM simulation of transverse effects in SAW resonators on lithium tantalate. IEEE Trans. Ultrason. Ferroelectr. Freq. Control.

[19] Laude V., Reinhardt A., Wilm M., Khelif A., Ballandras S. (2004). Fast FEM/BEM simulation of SAW devices via asymptotic waveform evaluation. IEEE Trans. Ultrason. Ferroelectr. Freq. Control.

[20] Hashimoto K.-Y., Koseki Y., Yamaguchi M. (1991). Boundary Element Method Analysis of Interdigital Transducers Having Arbitrary Metallisation Ratio. Jpn. J. Appl. Phys..

[21] Kochanov E.S. (1967). Parasitic capacitances in printed wiring of radio equipment. Telecommun. Radio Eng..

[22] Gevorgian S.S., Martinsson T., Linner P.L.J., Kollberg E.L. (1996). CAD models for multilayered substrate interdigital capacitors. IEEE Trans. Microw. Theory Tech..

[23] Igreja R., Dias C.J. (2011). Extension to the analytical model of the interdigital electrodes capacitance for a multi-layered structure. Sens. Actuators A Phys..

[24] Ghione G., Goano M. (2003). Revisiting the partial-capacitance approach to the analysis of coplanar transmission lines on multilayered substrates. IEEE Trans. Microw. Theory Tech..

[25] Crampagne R., Ahmadpanah M., Guiraud J.L. (1978). A simple method for determining the Green’s function for a large class of MIC lines having multilayered dielectric structures. IEEE Trans. Microw. Theory Tech..

[26] Hashimoto K.-Y. (2000). Surface Acoustic Wave Devices in Telecommunications.

[27] Liu B., Zeng Y. (2016). Uncertainty-aware frequency estimation algorithm for passive wireless resonant SAW sensor measurement. Sens. Actuators A Phys..

[28] Salzenstein P., Wu T.Y. (2023). Uncertainty Estimation for the Brillouin Frequency Shift Measurement Using a Scanning Tandem Fabry—Pérot Interferometer. Micromachines.

[29] Bergmann A., Waldherr A., Kirschner H.P., Wagner K. High selectivity SAW duplexer for W-CDMA Band VIII. Proceedings of the IEEE Ultrasonics Symposium 2008.

[30] Inoue S., Tsutsumi J., Matsuda T., Ueda M., Ikata O., Satoh Y. (2007). Ultra-Steep Cut-Off Double Mode SAW Filter and Its Application to a PCS Duplexer. IEEE Trans. Ultrason. Ferroelectr. Freq. Control.

[31] Inoue S., Tsutsumi J., Iwamoto Y., Matsuda T., Miura M., Satoh Y., Ueda M., Ikata O. 1.9 GHz range ultra-low-loss and steep cut-off double mode SAW filter for the Rx band in the PCS antenna duplexer. Proceedings of the IEEE Symposium on Ultrasonics 2003.

[32] Changzhou ChemSemi Co. Ltd. http://www.chemsemi.com/html/Technology/.

[33] Kawachi O., Mitobe S., Tajima M., Inoue S., Hashimoto K.-Y. (2007). Low-Loss and Wide-Band Double-Mode Surface Acoustic Wave Filters Using Pitch-Modulated Interdigital Transducers and Reflectors. IEEE Trans. Ultrason. Ferroelectr. Freq. Control.

